# Modified Tooth Key

**Published:** 1848-10

**Authors:** 


### MODIFIED TOOTH KEY.

A few weeks ago, we received from Dr. E. Bourne, Dental Surgeon, of Florence, Ala., a beautifully finished tooth key, made by himself, which will doubtless prove to be a useful instrument. The shaft is straight, tapers from the handle to its extremity, and is four inches and a quarter long. Its extreme length from the palmar surface of the handle to the extremity of the screw which retains the hook in its position, is five and a quarter inches. The fulcrum projects at right angles from the extremity of the shaft, a quarter of an inch, and possesses equal width and thickness,—the sides of the fulcrum are concave, so as to fit the convex surface of the teeth; while its lower longitudinal plane has a deep groove filed out, running parallel with it, the edges of which are serrated, exhibiting the appearance of a stationary claw on each side. There is but one groove, wherein to fasten a hook, and the bottom of which is flat, so that the body of the hook is intercepted by the outer edges of its floor, when it falls to that point in whieh the claw extremity approaches the groove in the fulcrum. Its action is intended to be an imitation of the Forceps, in preventing the laceration of the gums, yet, at the same time preserving the power of the key.

We will take this occasion to say to our Western and Southern brethren, that rare and interesting cases frequently occur in practice which the profession ought to be advised of:—that every practitioner is forced to contrive useful appliances, to meet pressing exigencies, in every department of the profession,—and that nothing can enrich the profession more than to give publicity to every thing which can, in any way, tend to advance the general interest. For this purpose the pages of the Register will be found the best medium through which to disseminate useful knowledge in Dental Surgery throughout the West and South.—St. Louis Ed.

